# Editorial: Investigation of brain functional connectivity from electroencephalogram data

**DOI:** 10.3389/fphys.2022.1058683

**Published:** 2022-10-31

**Authors:** Giovanni Chiarion, Soroush Safaei, Alireza Valizadeh, Pouya Bashivan, Chien-Hung Yeh, Chuting Zhang, Yufei Wang, Luca Mesin

**Affiliations:** ^1^ Mathematical Biology & Physiology, Department Electronics and Communications, Politecnico di Torino, Turin, Italy; ^2^ Auckland Bioengineering Institute, University of Auckland, Auckland, New Zealand; ^3^ Department of Physics, Institute for Advanced Studies in Basic Sciences (IASBS), Zanjan, Iran; ^4^ Pasargad Institute for Advanced Innovative Solutions (PIAIS), Tehran, Iran; ^5^ Department of Physiology, McGill University, Montreal, QB, Canada; ^6^ School of Information and Electronics, Beijing Institute of Technology, Beijing, China

**Keywords:** brain, functional connectivity, EEG, electroencephalogram, graph theory, machine learning

Neuroimaging and electrophysiological recordings have revealed coordinated activity of anatomically separated brain regions. Several brain functions are dependent on the integration of the information processed in functionally specialized regions and the coordination between the activities of the brain regions controls the communication and integration of information in the brain circuits (Fries, 2005). A variety of methods are used to determine the functional connectivity (FC), which is constructed upon statistical interrelations between brain regions and quantifies communication and the functional similarity between them (Park and Friston, 2013). Consequently, study of the functional networks is deemed crucial for understanding the mechanisms of brain-wide information processing in healthy brains (Nentwich et al., 2020) and for developing treatments for many of the most complex brain disorders (Chiarion and Mesin, 2021). FC methods span a variety of disciplines including mathematics, computer science, signal processing, medicine, neuroscience, physiology, physics, electrophysiology, and psychology.

Functional connectivity is usually inferred from the correlation in the activity of the nodes based on the BOLD or synchrony in the EEG or MEG signals. The present Research Topic focuses on the investigation of FC using electroencephalography (EEG) that, thanks to its high temporal resolution, enables recording brain activity patterns that could appear in short timescales and allows to conduct spectral analysis, which is not possible with BOLD signals. On the other hand, EEG signal is often noisy and highly affected by volume conduction and common source issues. For this reason, appropriate methodologies for processing the EEG data are of utmost importance, to assure reliable evaluation of the FC measurements (Shi et al., 2019).

The articles in the current Research Topic investigate various applications, using different approaches. The following is a brief introduction to the contributions.

1. Kaminsky and Blinowska, who had previously proposed the widely used Direct Transfer Function (DTF) metric, provide a guide toward future research in FC analysis in EEG. In particular, their paper analyzes different FC approaches (e.g., linear vs nonlinear, bivariate vs multivariate methods, etc.) with respect to the effect that these choices have on addressing issues due to noise, volume conduction, common drive, etc. Characterization of networks is also treated with critical analysis of the definition of “smallworldness”.
2. Song et al. focuses on the analysis of the different brain organization of depressed patients during sleep with respect to controls. The high temporal resolution of the EEG is exploited with dynamic FC analysis (dFC), using the weighted phase lag index (wPLI) as a bivariate metric and dividing the signal epochs by sleep stages and frequency bands. Interestingly, they found that depressed patients exhibit higher node strength and global efficiency than controls in all frequency bands and stages of sleep.
3. Jin et al. applies the adaptive frequency band division to the analysis of gait disturbance in Parkinson’s disease (PD). Cross-frequency coupling (CFC) techniques are used to investigate functional connections under the dynamic gait phase. In particular, the authors evaluated phase-amplitude coupling (PAC), exploring the relationships between the amplitude of high-frequency rhythms (i.e., *β* and *γ*) and the phase of low-frequency rhythms (i.e., main rhythms of lower frequency) of local field potentials in the subthalamic nucleus (STN) in PD patients. As an important result, they revealed that there is a suppression of exaggerated *β*/*γ*-band-related PAC and an enhanced gait-phase-related modulation of the high portion of *β* in the STN, corresponding to the improvement of stepping performance using auditory cues.
4. Ho et al. applies the global synchronization (GFS) metric to predict the outcome of sudden cardiac arrest (SCA) survivors. Since GFS is known to be influenced by noise, they proposed an anti-noise algorithm that reduces its effect showing that patients with a good outcome (GO) have a higher EEG power in all the four analyzed frequency bands (i.e., *δ*, *θ*, *α* and *β*). Using the severity-of-disease classification system APACHE II, they also found that *α* band is the most useful in predicting GO in SCA survivors.
5. Pirovano et al. highlights the importance of assessing the directionality of the information flows in the FC analysis of stroke patients after rehabilitation. They propose to combine the information of direct and indirect coupling provided by two frequency-domain measures: directed coherence (DC) and generalized partial directed coherence (gPDC). They observed a significant increase in the topographic representation of intra-hemispheric connections as well as an increase in power transfer. In particular, in the *α* frequency band, they found an improvement in information flow between the pre-motor cortex (pMC) and primary motor cortex (M1) areas in the ipsi- and contra-lesional hemispheres. As a final result, they found a significant increase in the topographic representation of the connection from supplementary motor area (SMA) to contra-lesional M1 in *α* frequency, while a decrease in the contra-lesional pMC → SMA coupling after rehabilitation was observed both in *α* and *β* frequency bands. Considering only the DC, they would not be able to identify changes in SMA connections with contra-lesional motor and pre-motor areas, as well as the enhanced role of the pMC inter-hemispheric coupling in the *β* band.

In summary, this Research Topic presents novel approaches to various applications of FC analysis on EEG data. Different FC metrics can be useful for exploring brain network connections under different physiological or pathological conditions. Many more applications are expected in the future.

## References

[B1] Chiarion G. Mesin L. (2021). Functional connectivity of eeg in encephalitis during slow biphasic complexes. Electronics 10, 2978. 10.3390/electronics10232978

[B2] Fries P. (2005). A mechanism for cognitive dynamics: Neuronal communication through neuronal coherence. Trends Cogn. Sci. 9, 474–480. 10.1016/j.tics.2005.08.011 16150631

[B3] Nentwich M. Ai L. Madsen J. Telesford Q. Haufe S. Milham M. (2020). Functional connectivity of eeg is subject-specific, associated with phenotype, and different from fmri. Neuroimage 218, 117001. 10.1016/j.neuroimage.2020.117001 32492509 PMC7457369

[B4] Park H. Friston K. (2013). Structural and functional brain networks: From connections to cognition. Science 342, 1238411. 10.1126/science.1238411 24179229

[B5] Shi W. Yeh C. H. Hong Y. (2019). Cross-frequency transfer entropy characterize coupling of interacting nonlinear oscillators in complex systems. IEEE Trans. Biomed. Eng. 66, 521–529. 10.1109/TBME.2018.2849823 29993517

